# Facial Line Angles: A Key to Tooth-like Rehabilitation

**DOI:** 10.1155/2022/4917536

**Published:** 2022-10-12

**Authors:** Rim Kallala, Yosra Gassara, Ines Azouzi, Amani Adli, Dalenda Hadyaoui, Zohra Nouira, Chiraz Baccouche, Soumaya Touzi, Belhassen Harzallah, Mounir Cherif

**Affiliations:** ^1^University of Monastir, Faculty of dental Medicine, Department of dental anatomy, Monastir, Tunisia; ^2^University of Monastir, Research Laboratory of Occlusodontics and Ceramic Prostheses LR16ES15, 5000 Monastir, Tunisia; ^3^Department of Fixed Prosthodontics of the Dental Clinic of Monastir, Monastir, Tunisia; ^4^Department of Fixed Prosthodontics of the Military Hospital of Instruction of Tunis, Tunis, Tunisia

## Abstract

Recently, improvement of appearance and a quest for beauty have become a primary concern for patients. It is a challenging task for a clinician to achieve esthetic integration of prosthetic pieces for anterior teeth, particularly for highly demanding patients who give attention to particular details. The challenge is harder when only one tooth has to be restored. The objective is to achieve a fully and perfectly integrated rehabilitation with natural dentition. Poorly described, facial line angles are key to the success of achieving the desired tooth shape, especially for the maxillary central incisor. They influence both the shape and color of the tooth through optical illusion. Their misplacement could certainly spoil the esthetic outcome. Thus, it is mandatory to respect and recreate them. The objective of the present study was to define and then to highlight their importance. It also aimed to give some tips on how to perfect the shape of prosthetic teeth through a clinical case of central incisor esthetic rehabilitation.

## 1. Introduction

Recently, improvement of appearance and a quest for beauty have become a primary concern of patients. Esthetic dentistry is one of the most concerned fields by this vogue. The smile is a means of expression and communication. Having a perfect one is a purpose, nowadays [1]. For anterior teeth, it is a challenging task for a clinician to succeed esthetic integration of prosthetic pieces, particularly for highly demanding patients who give attention to particular details [1]. This challenge is harder when only one tooth has to be restored. Here, the purpose is to achieve unnoticed and unremarkable rehabilitation. Poorly described, facial line angles are key to the success of achieving the desired tooth shape [2], especially for the maxillary central incisor. Their misplacement could certainly spoil the esthetic outcome. Thus, it is mandatory to respect and recreate these lines during wax-up and prosthesis design [2]. The objective of the present paper was to define and then to highlight their importance. It also aimed to give some tips to perfect the shape of prosthetic teeth through a clinical case of central incisor esthetic rehabilitation.

## 2. Definition and Features

Line angles are defined as the lines that mark the transition from the buccal and lingual surface to the proximal one [2]. Each tooth exhibits four lines angles: mesiofacial, distofacial, mesiopalatal, and distopalatal.

**Figure 1:**
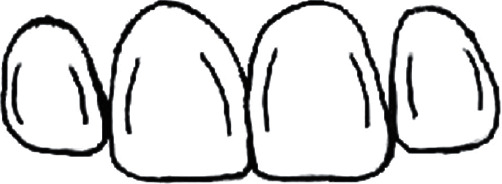
Facial Line angles symmetry with contralateral tooth.

From a morphological point of view, these lines are formed by the conjunction of the cervical line to the contact area [3]. They obey the symmetry rule in regard to a straight line on the contact zone [3]. Consequently, the mesial transition line is symmetrical in regard to the distal one of the proximal teeth [3] (Figure 1). Interacting with each other, line angles create both cervical and facial embrasures [3, 4].

**Figure 2:**
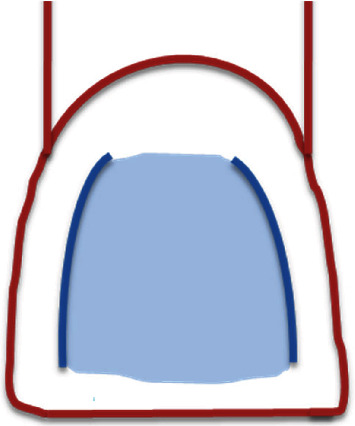
Tooth facial reflexive surface bounded by mesial and distal line angles.

According to Sesemann, a line angle is defined as the line at which two planes intersect [4]. Geographical size and magnitude of both reflexive and deflective surfaces are defined by the tooth line angle. Thus, the visual perception the tooth labial surface is significantly dependent on the teeth (Figure 2). When viewed from the frontal plane, both mesial and distal line angles are slightly curved, whereas, when viewed from the sagittal plane, they are straight [4].

## 3. Clinical Presentation

A 31-years-old healthy woman consulted the Fixed Prosthetic Department of the Clinical Dentistry of Monastir, Tunisia. The patient asked to replace her prosthetic upper-right central incisor. She was unsatisfied, especially with the shape of the tooth.

**Figure 3:**
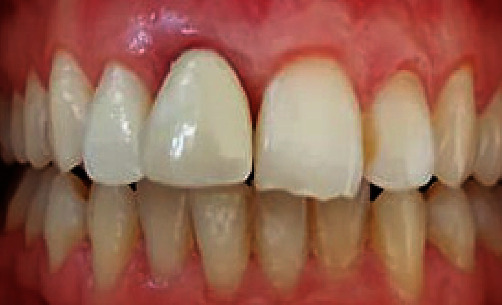
Initial situation: The upper right central incisor is crowned and displays triangular shape.

The intraoral examination showed that the crowned tooth exhibited triangular shape, while the contralateral one was oval (Figure 3). An evident over-contour, spoiling the smile, was responsible of the plaque retention at that area. Both edges were not aligned: the one of the left central incisor was irregular (Figure 3). The patient reported that her two incisors were fractured when she was young.

**Figure 4:**
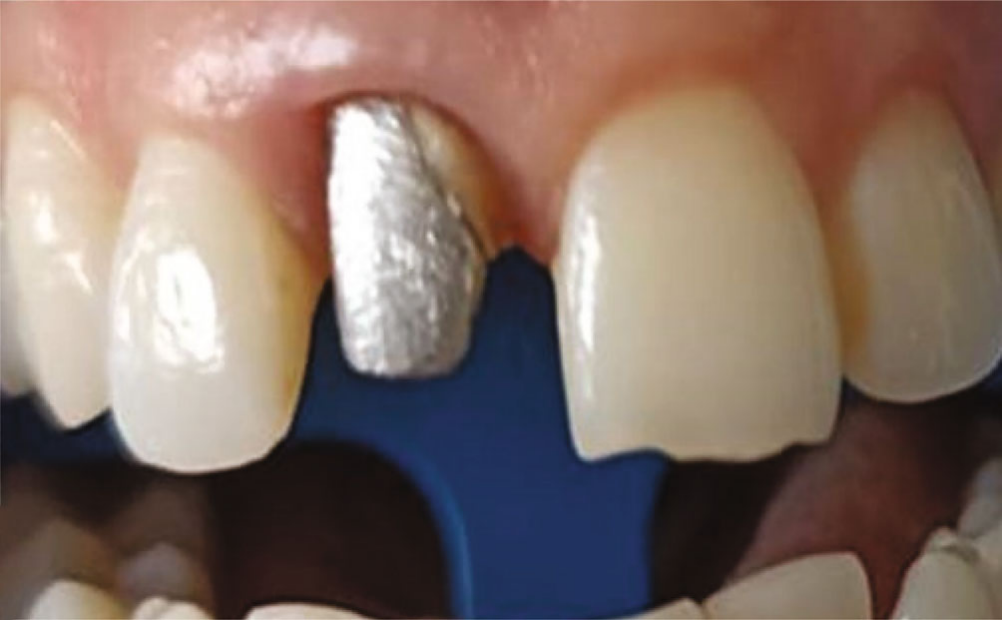
After the cementation of the metallic post and core.

**Figure 5:**
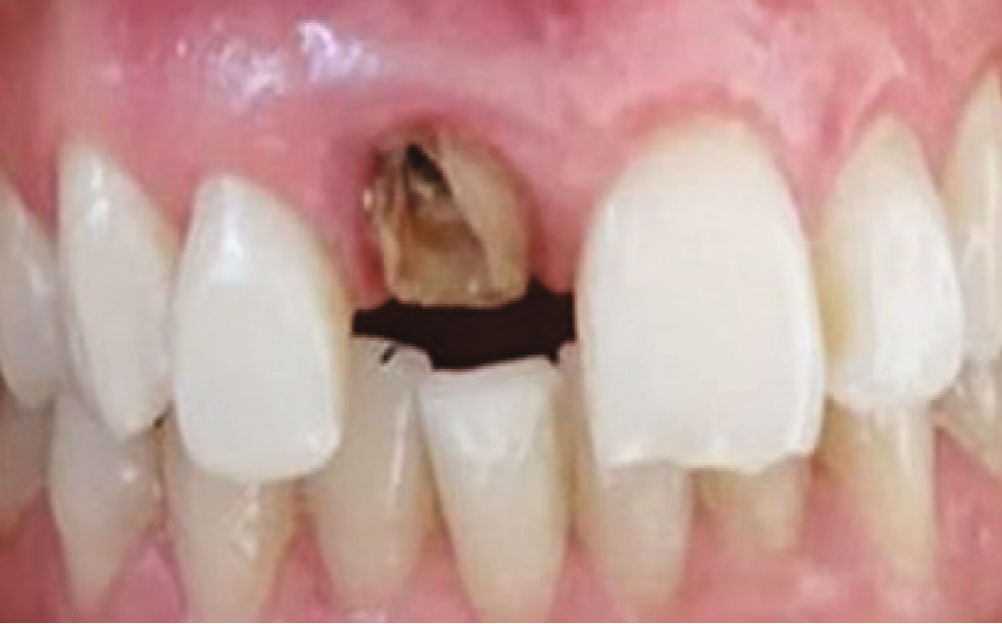
After the crown removal: dental tissue loss was subtotal and margins were deep.

The first step was the crown removal. The loss of dental tissue was very important, and the abutment did not have enough walls to provide an adequate ferrule effect [5] (Figure 4). For this reason, the orthodontic extrusion was proposed to the patient [5]. Because of a lack of time, she refused this alternative. So, she was clearly informed that lack of a ferrule tooth structure may affect the abutment's strength resistance to fracture [5]. Thus, the metallic post and core was the most suitable solution due to its better fracture resistance than prefabricated glass fiber posts [6]. Once the informed consent to the treatment was obtained, it was carefully placed (Figure 5).

The zirconia material was chosen in order to hide the metallic post and core color.

**Figure 6:**
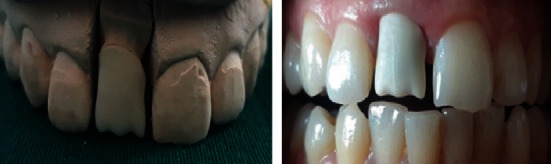
The checking of zirconia framework.

**Figure 7:**
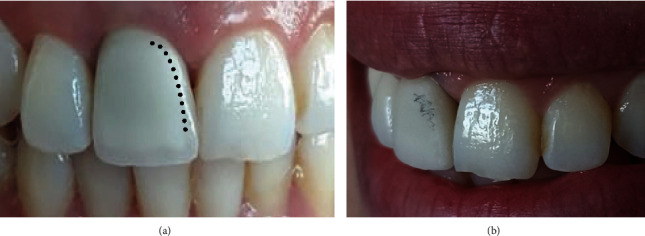
The intra oral checking of the crown after ceramic stratification showing inappropriate mesial line angle (a) from the frontal view and (b) from the lateral view.

**Figure 8:**
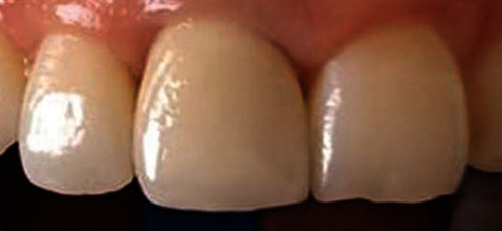
The shape improvement after line angle displacement.

**Figure 9:**
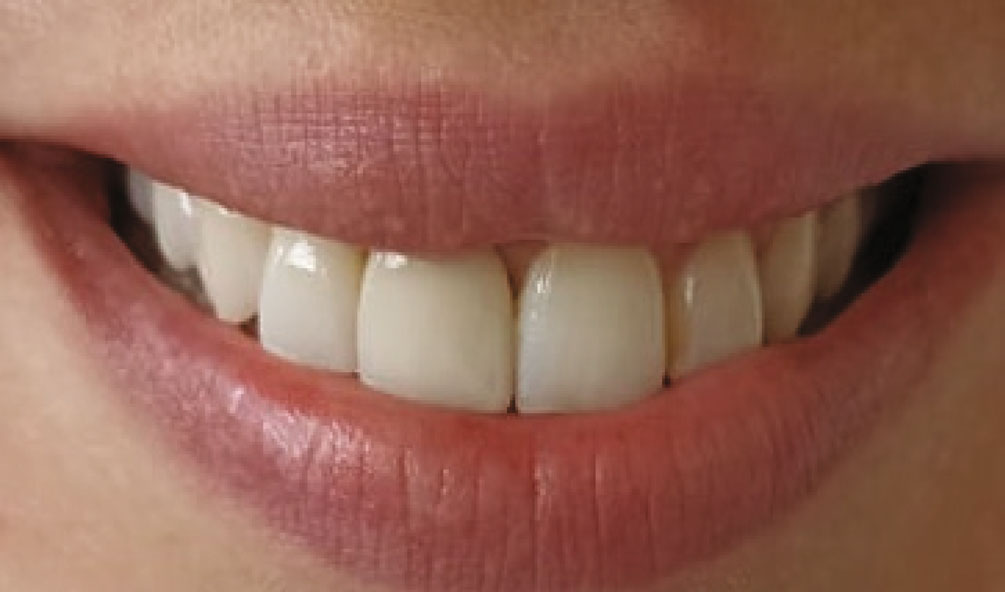
Final outcome showing unnoticed rehabilitation.

The framework was checked, intraorally (Figure 6), and the space left for ceramic was validated (Figure 6). Then, it was sent to the laboratory to perform the ceramic stratification. Once received, the insertion and cervical adaptation of the prosthetic crown were checked. The tooth shape was also carefully evaluated. The crown shape was triangular compared with the oval homologue one. The mesial line angle was regulated, and the shape was optimized. As the patient was anxious about her teeth, the incisal edge of left central incisor was just regularized (Figures 7 and 8). After glazing, the esthetic outcome was pleasant and the patient was satisfied (Figure 9).

## 4. Discussion

The American Academy of Cosmetic Dentistry has included line angles among the 44^th^ criterion used by accreditation examiners to assess clinical cases submission [4]. They emphasized the fact that facial morphology of prosthetic rehabilitation have to mimic, at most, the natural one. Authors consider that the tooth's perceived [7] dimensions are those measured between mesial and distal line angles, which is called the virtual width [7]. Others attributed them to the apparent tooth width, apparent face width, and perceived tooth width [7, 8]. Some others recommended the use of the distance between facial line angles as a tool to check the tooth size and symmetry [8].

Understanding the tooth morphology is considered the first key step to achieving esthetic integration. It is important to recognize that line angles are anatomical structures that provide the tooth shape, which is called the primary anatomy [9]. Their ignorance could lead to bulky shape and under-contoured proximal areas. That is why drawing them directly on the restoration seems to be useful (Figure 7).

Mainly, three shapes of maxillary central incisors are described: triangular, oval, and square. According to Paulo Monteiro [9], four guidelines are essential to define the tooth shape: the outline silhouette, lines angles or primary anatomy, macro texture or secondary anatomy and micro texture or tertiary anatomy.

**Figure 10:**
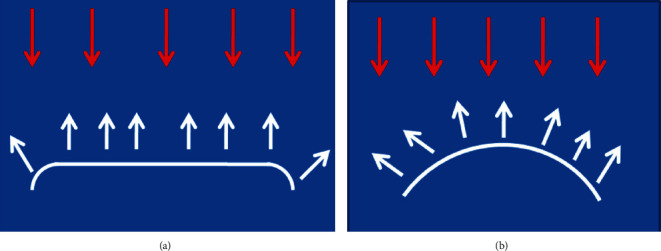
The light incidence on tooth surface leading to optical illusion [10]. (a) Plate surface. (b) Rounded surface.

Due to facial line angles, clinician could display on tooth dimension, and it is called illusion phenomenon [2, 4]. Thus, the area between them, when light strikes directly, is reflected back [2]. However, the outside area deflects less the light and consequently, it is less noticeable (Figure 10). This feature is used, generally, to increase or decrease the tooth width [10] (Figure 10).

The improper placement of facial line angles would make the restoration or the prostheses appear different, although it exhibited the same size of the contralateral tooth [2]. The closer the lines to the tooth's center are, the narrower the tooth appears, while the further the lines to the tooth's center are, the wider the tooth would appear [4, 7].

Also, rounded surface produces optical width reduction. However, plate surface increases it (Figure 10) [10].

Also, when tooth anatomy is not respected, the light deflection is different from the that of the contralateral tooth. Consequently, both the shade and shape would be inappropriate [4, 7].

Sesemann [4] has reported that the 20 square millimeters in smile design occurs between mesiofacial line angles of the central incisors at proximal contact area. He added that when they are bilaterally asymmetrical, the midline would appear canted even when it was perfectly parallel to the face midsagittal midface [4]. The reported case showed the midline discrepancy at the stage of ceramic trying (Figure 7a). The simple removal of the mesial line angle was sufficient to optimize the shape and to recreate a respected midline between central incisors (Figure 8).

The clinician has to carefully evaluate the tooth shape and line placement at the stage of ceramic trying. Embrasures and incisal edge, have to be checked as well. All these characteristics have to be checked from different views (Figure 7).

On the other hand, for fixed monolithic milled prostheses, the innovation of biogeneric model could solve such a problem. This innovative tool, based on mathematic algorithms, would give the ideal tooth morphology [11]. Various studies were conducted comparing the biogeneric tooth morphology with natural ones [11]. Others have compared them with wax done by a laboratory technician [12]. They affirmed the contribution of the biogeneric model in both morphology and occlusion improvement.

## 5. Conclusions

The tooth morphology understanding is the first step to the success of esthetic dentistry for both composite restoration and fixed prostheses. The placement of line angles is crucial to achieving a natural outcome. Respecting the symmetry is very useful. For milled restorations, the biogeneric model could facilitate their creation and respect. The clinician has to be wise and attentive to achieve the esthetic outcome [10].
